# Laterality of deep white matter hyperintensities correlates with basilar artery bending and vertebral artery dominance

**DOI:** 10.3325/cmj.2021.62.360

**Published:** 2021-08

**Authors:** Laszlo Szalontai, Zsofia Jokkel, Tamas Horvath, Bianka Forgo, Ildiko Kalina, Pal Maurovich-Horvat, Philip L. Auyang, M. Mujeeb Zubair, Zsolt Garami, David Laszlo Tarnoki, Adam Domonkos Tarnoki

**Affiliations:** 1 Medical Imaging Centre, Semmelweis University, Budapest, Hungary; 2 Department of Hydrodynamic Systems, Budapest University of Technology and Economics, Budapest, Hungary; 3 Department of Radiology, Örebro University Hospital, Örebro, Sweden; 4 Department of Cardiovascular Surgery Methodist De Bakey Heart & Vascular Center, Houston, Texas

## Abstract

**Aim:**

To investigate whether vertebrobasilar geometry contributes to the presence, severity, and laterality of white matter hyperintensities (WMH).

**Methods:**

We retrospectively reviewed 290 cerebral scans of patients who underwent time-of-flight and fluid-attenuated inversion recovery (FLAIR) magnetic resonance imaging (MRI) between 2017 and 2018. WMH were counted, localized, and grouped according to laterality on the FLAIR sequence. A 3D mesh of the posterior circulation was reconstructed (with ITK SNAP software) and the morphology of the vertebrobasilar system analyzed with an in-house software written in Python.

**Results:**

Patients were assigned into a group with WMH (n = 204) and a group without WMH (n = 86). The severity of WMH burden was mainly affected by age and hypertension, while the localization of the WMH (or laterality) was mainly affected by the vertebrobasilar system morphology. Basilar artery morphology only affected the parieto-occipital region significantly if both posterior communicating arteries were hypoplastic or absent. The dominant vertebral artery and basilar artery curve had an opposite directional relationship.

**Conclusions:**

An unequal vertebral artery flow is an important hemodynamic contributor to basilar bending. Increased basilar artery curvature and increased infratentorial WMH burden may signal inadequate blood flow and predict cerebrovascular events.

Cerebrovascular diseases, predominantly stroke, remain the leading cause of death and functional disability worldwide (1). White matter hyperintensities (WMH) on cerebral T2-weighted fluid-attenuated inversion recovery (FLAIR) magnetic resonance imaging (MRI) sequences have been linked to cerebrovascular disease outcomes and ischemic stroke (2,3) (Figure 1). WMH, subclinical cerebral alterations caused by changes in cerebral vascular morphology, may lead to cerebral histological changes long before the manifestation of neurological deficits or cognitive decline (4). Multiple studies suggested that ischemia plays a crucial role in the development of tortuous arterioles and reduced cerebral blood flow. Despite the two decades of advancements in the understanding of the etiology of these processes, much uncertainty remains (2). Chronic hypoperfusion and arteriolosclerosis are considered key features in the development of WMH. The presence of WMH triples the risk of stroke and doubles the risk of dementia (5). Previous publications showed a significant correlation between cerebral artery morphology and stroke localization (6). However, the connection between WMH and large cerebral artery morphology is not yet fully understood.

**Figure 1:**
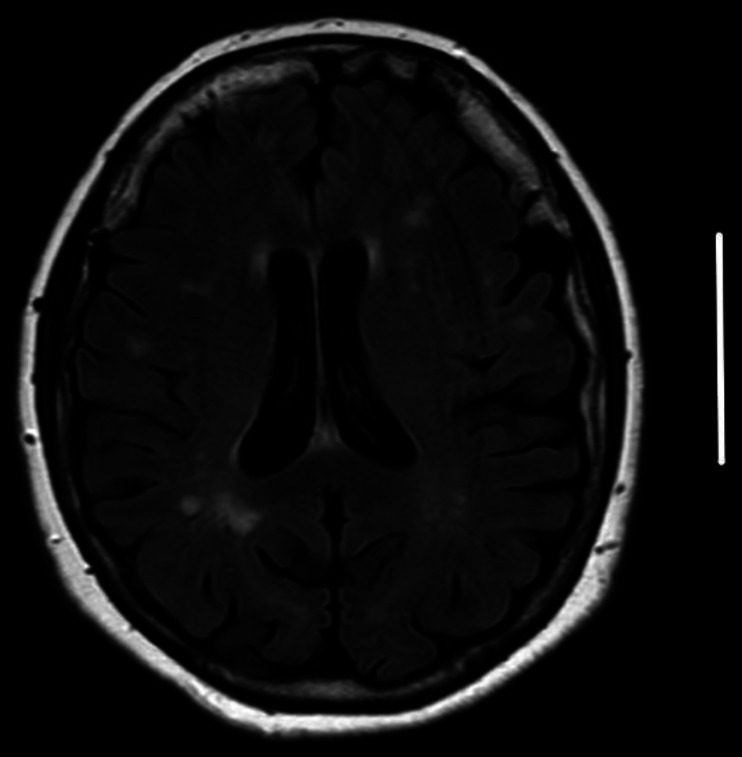
White matter hyperintensity on T2 fluid-attenuated inversion recovery magnetic resonance imaging sequence (scale bar, 5 cm; source: Medical Imaging Centre, Semmelweis University). Based on our white matter hyperintensities evaluation protocol, this patient was categorized as posterior cerebral artery region right dominant.

Approximately 20% of strokes occur in the region supplied by the vertebrobasilar system. Unlike most systemic arteries, which are characterized by a tree-like branching pattern, the basilar artery is unique as it is created from the confluence of the two vertebral arteries. The vertebral arteries are mostly asymmetric and left-dominant. The varying vessel morphologies may cause asymmetrical blood flow in the basilar artery, which over time possibly leads to bending. This phenomenon, named basilar curve or basilar bending, may create microvascular damage to the surrounding brain areas.

Therefore, our first aim was to investigate if basilar artery geometry affected the existence and laterality of WMH. Our second aim was to investigate the influence of the vertebral artery dominance on basilar artery geometric indices.

## Methods

### Patients

We retrospectively reviewed the scans of patients who underwent time of flight (TOF) and FLAIR MR imaging (Philips Ingenia 1.5T, Philips, Amsterdam, the Netherlands) at the Medical Imaging Centre, Semmelweis University, between 2017 and 2018. Most patients were referred from the Department of Neurology due to visual impairment, headache, or dizziness. Information on comorbidities such as hypertension, hyperlipidemia, and diabetes mellitus was gathered from medical charts. The exclusion criteria were medical history of large vessel obstruction, stroke, vasculitis, demyelinating disease, brain malignancies, abscess, or encephalitis as these diseases may also present with WMH. Patients with autoimmune diseases and migraine were also excluded, as well as patients younger than 20 years since the development of the basilar curve is a highly age-related and long-term process.

### Imaging analysis

Images were assessed by a single reader blinded to the clinical information. Based on FLAIR images (echo time [TE] 140 ms, repetition time [TR] 9000-11 000 ms, inversion time 2450-2800 ms, slice thickness 5 mm), the patients were assigned to a group with WMH and a group without WMH. On WMH scans, the brain was divided into a right and left side by a virtual vertical line along the falx cerebri. Each side was also divided into white matter regions supplied by large vessels (vertebral – VA, basilar – BA, and posterior cerebral artery – PCA) (Table 1). These regions were identified by means of templates based on imaging and anatomical studies (7). The regions were evaluated separately according to age-related white matter changes (ARWMC) scale (8). Each region’s WMH burden was compared with that on its opposite side. The dominant WMH side (laterality) was identified either by a higher ARWMC score, or if the score was the same on both sides by manual counting of the WMH lesions that were ≥5 mm (8) (Figure 1). The posterior communicating arteries were also analyzed, but due to the restrictions of the 1.4-mm MRI slice thickness they were categorized as either “hypoplastic/no flow” or “flow detected.”

**Table 1:** Regions of interest supplied by the vertebral, basilar, and posterior cerebral artery created with anatomical templates (7)

Region	Territories involved
Vertebral artery region	Lower 2/3 of the cerebellum and medulla
Basilar artery region	Upper 1/3 of the cerebellum and pons
Posterior cerebral artery region	Areas of the parieto-occipital lobe

### 3D vascular reconstruction

**Figure 2:**
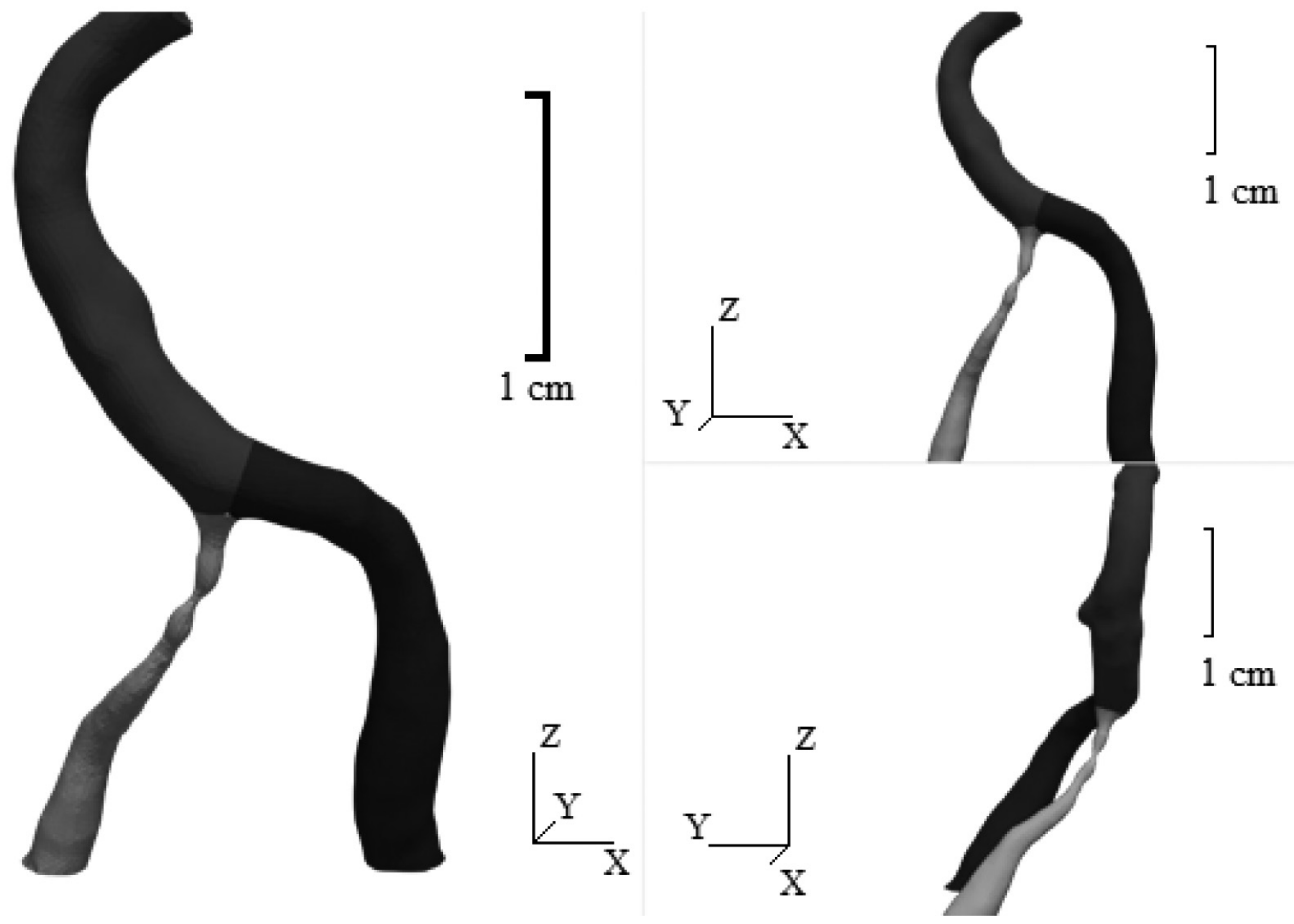
Three-dimensional reconstruction of the two vertebral arteries and the basilar artery made with the ITK-SNAP software, version 3.8.0.

**Figure 3:**
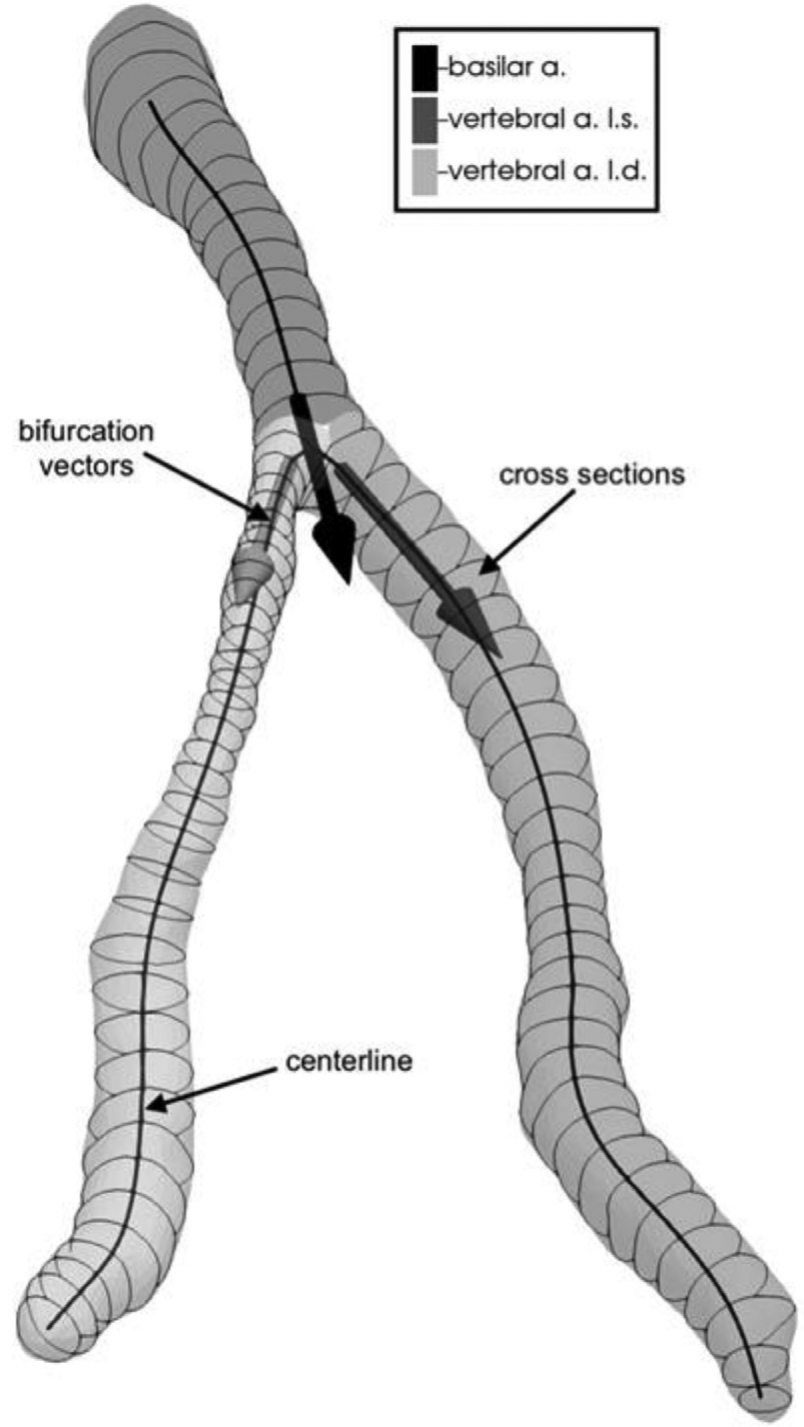
Visual representation obtained by an in-house software analyzing the vertebrobasilar reconstruction of a patient. The program identifies the arteries based on the confluence of the two vertebral arteries, creates the centerline for each vessel, and measures different geometrical indices. Basilar a. – basilar artery, vertebral a.l.s. – left vertebral artery, and vertebral a.l.d. – right vertebral artery.

We used TOF angiography MRI (TE 3-7 ms, TR 18 ms, flip angle 20°, slice thickness 1.4 mm) to visualize the flow within the vessels, without the need to administer contrast agent. To ensure objective, repeatable, standardized, and automated measurements we reconstructed the vertebrobasilar vascular system based on this sequence with the ITK-SNAP software (version 3.8.0.), which is the gold standard for modeling arteries (9) (Figure 2). In each reconstruction, a lower (approx. 850 ± 50 gray level intensity) and upper (approx. 2500 gray level intensity) threshold were set and reconstructed with a built-in automated segmentation tool. As artifacts may appear during the reconstruction process, the models were smoothed with the Taubin algorithm using MeshLab, version 2016.12. Thus, we obtained the same three main arteries to ensure standardized measurement (10). We then created an automated measurement method with an in-house application written in Python (scripts are available from the corresponding author on request). This program automatically recognizes the left and right vertebral artery and basilar artery and provides standardized measurements that can be used in the analysis of morphological properties of these arteries, ensuring the method's repeatability (Figure 3). Difference in diameter of 0.3 mm or more between the two vertebral arteries was used to define the dominant artery, based on earlier publications (11), and to eliminate artifact-based differences. The volume, curvature, torsion, and tortuosity of both vertebral arteries were also assessed, as well as the length, volume, curvature, and cross-section of the basilar artery. We connected the confluence of the vertebral arteries and the basilar top with a straight line and grouped the patients based on if the basilar artery deviated to the left or to the right, while also measuring the deviation extent.

### Statistical analysis

Differences between the WMH and control group were assessed with the Mann- Whitney-U test. The Fisher exact test was used to compare WMH laterality with vertebral artery dominance and basilar artery curvature. The Spearman test was used to assess the directional relationship between the vertebral artery and the basilar artery based on their morphology. Logistic regression analysis was performed for lesions in the PCA regions to determine the predictors of WMH. Bonferroni correction was applied. *P* values of less than 0.001 were considered significant. Statistical analysis was performed in R studio, version 1.1.463 and SPSS, version 24 (IBM Corp, Armonk, NY, USA)

## Results

The study enrolled 332 patients who underwent TOF and FLAIR MRI (Table 2); 42 patients were excluded. The average age of the remaining 290 patients (59.3% female) was 52.4 ± 17.6 years. The WMH group consisted of 204 patients (average age 57.6 ± 16.7 years; 57.8% female) and the control group consisted of 86 patients (average age 40.2 ± 13.0 years, 62.0% female). The WMH group had significantly higher median age (62 years vs 37 years, U = 3681, z = 7.67, *P* < 0.001, r = 0.45) and a greater frequency of hypertension and diabetes mellitus compared with the control group (*P* = 0.004, odds ratio [OR] = 8.75 and *P* < 0.001, OR = 4.08, respectively, Fisher exact test).

**Table 2:** Demographic data and risk factors in the white matter hyperintensities (WMH) group and controls

	Total (n = 290)	Control group (n = 86)	WMH group (n = 204)
Demographic data			
Age (years, mean ± standard deviation)	52.4 ± 17.6	40.2 ± 13.0	57.6 ± 16.7
Sex (male/female)	118/172	32/54	86/118
Risk factors, N (%)			
Hypertension	126 (43)	11 (13)	115 (56)
Diabetes mellitus	38 (13)	4 (5)	34 (17)
Hyperlipidemia	18 (6)	4 (5)	14 (7)

As expected, our patients more commonly had left vertebral artery dominance (Table 3). In contrast, vertebra artery curvature (median = 0.15, U = 3980, z = 3.9, *P* < 0.001, r = 0.26) and torsion (median = 4.9, U = 4757, z = 2 *P* = 0.002, r = 0.13) were significantly greater in the non-dominant vertebral artery. The WMH burden in the VA region was significantly higher in the non-dominant side of the vertebral artery (*P* = 0.006, OR = 0.13, Fisher exact test). Sixty-six patients had less than 0.3 mm difference between the two vertebral arteries' cross-sections and were classified as even. These patients showed no correlation with cerebellar WMH laterality.

**Table 3:** Side of the dominant vertebral artery in the white matter hyperintensities (WMH) group and controls. The values are counts

Side of dominant vertebral artery	Total (n = 290)	Control group (n = 86)	WMH group (n = 204)
Right side (0.3 mm difference)	84	26	58
Left side (0.3 mm difference)	139	39	100
Classified as even	66	20	46

Vertebral artery dominance inversely correlated with basilar artery curve laterality (*P* < 0.001, r = -0.56, Spearman test), which has already been reported in previous publications (6,10). Three times more patients in the whole cohort had the right-curved basilar artery and left-dominant vertebral artery (Table 4). Tortuosity (median = 0.05%, U = 7253, z = 2.3, *P* = 0.019 r = 0.13), length (median = 24.13, U = 6897, z = 2.68, *P* = 0.005, r = 0.15), and mean cross-sectional area of the basilar artery (median = 10.73, U = 7013, z = 2.69, *P* = 0.007, r = 0.15) were significantly greater in the WMH group. WMH dominance in the BA region was significantly more frequent on the opposite side of the basilar artery curve (*P* = 0.002, OR = 0.06, Fisher exact test).

**Table 4:** Directions of the basilar artery curve in the white matter hyperintensities (WMH) group and controls. The values are counts

Direction of basilar artery curve	Total (n = 290)	Control group (n = 86)	WMH group (n = 204)
Right curve	143	36	107
Left curve	41	7	34
Classified as even	106	43	63

Logistical regression that included the whole cohort showed age (b = 0.56 *P* < 0.001 OR = 1.06) and hypertension (b = 1.33 *P* < 0.001 OR = 1.23) to be predictors for WMH burden severity in the PCA region. Out of the 290 patients, 72 patients’ posterior communicating artery was detectable on both sides (25%); 56 patients (19%) had left non-detectable and 67 patients (23%) had right non-detectable posterior communicating arteries. Ninety-five patients (33%) had non-detectable posterior communicating arteries on both sides. Out of these 95 patients, 73 had WMH in the PCA regions. Logistic regression including these 73 patients revealed basilar deviation from the centerline (b = 0.316, *P* = 0.013, OR = 1.37) as a predictor for WMH laterality in the PCA region.

## Discussion

Our aim was to investigate whether T2 FLAIR WMH of the brain, which are a risk factor for stroke, are influenced by the morphological properties of the vertebrobasilar arteries. Although the relationship between vertebral artery dominance and basilar artery curvature has already been investigated, most of these studies focused on the connection with stroke occurrence and post-stroke outcomes (12).

As expected, more patients in our study had left-dominant vertebral artery, which can be linked to the left subclavian artery branching directly from the aortic arch (13). Our results show that one-side vertebral artery hypoplasia leads to basilar artery curving on the opposite side, which is consistent with previous reports (12). This finding may be explained by inconsistent wall shear stress on the vessel wall caused by decades of turbulent blood flow. These effects can increase the risk for atherosclerosis on the small curve of the basilar artery (10). Patients in the WMH group had an increased length, tortuosity, and volume of basilar arteries, which was also observed in patients with dolichoectasia (14). Intracranial arterial dolichoectasia is defined as an increased diameter (ectasis) and/or long and tortuous course (dolichos) of at least one cerebral artery and is considered an independent risk factor for stroke (15). However, the diagnosis of dolichoectasia requires the basilar artery to have a cross-section of more than 4.5 mm, and the values in all our patients were below this cut-off (16). Our results showing that age, hypertension, and diabetes increase the risk for WMH agree with the results of previous studies (17). The average age of our WMH patients was 57.6 ± 16.7 years. In this age range, approximately 20% of the population has WMH lesions, which by the age of 90 can amount to almost 100% (18). Strassburger et al (19) showed a significantly higher WMH burden in hypertensive patients compared with normotensive age-matched patients. Hyperintense lesions are caused by either small subcortical infarcts or more often by a process of incomplete infarction, which reflects chronically reduced blood flow caused by morphological changes in the vessels and arteriolosclerosis (2). In the VA region, we found a significantly higher WMH burden, increased vessel curvature, and torsion on the non-dominant side of the vertebral artery. Different vertebral artery flows have been linked to posterior inferior cerebellar artery territory infarctions in the hemisphere of the non-dominant vertebral artery (6,11,12). BA-region WMH burden was higher on the opposite side of the basilar artery curve. This might be a consequence of lower blood perfusion in the perforating arterioles branching from the small curve of the basilar artery or of micro-thrombi created by the oscillating turbulent flow. Basilar artery curving has also been linked to pontine infarction in the hemisphere opposite to the basilar artery curve (6,11,12). These findings suggest a stronger etiological connection between sub-tentorial WMH and the strokes manifested in these regions. WMH severity has been linked to an increased risk of stroke and higher cognitive decline after stroke (4). Jeerakathil et al (20) found that WMH was strongly related to Framingham stroke risk profile and its component risk factors. According to Fu et al (21), patients with high WMH burden had an increased risk of deep subcortical stroke and a recurrent stroke, but the authors did not assess whether the laterality of WMH correlated with stroke laterality. We found no connection between WMH severity in any region as assessed with the ARWMC scale and basilar artery curvature and vertebral artery diameter.

Expectedly, in the PCA regions age and hypertension were identified as independent risk factors of WMH. However, none of the basilar morphological indices was significantly associated with the severity of WMH. This can be explained by posterior communicating arteries providing anastomoses to the vertebrobasilar system and decreasing the effects of basilar artery morphology. When only patients with two-sided posterior communicating artery hypoplasia/occlusion were included in the logistic regression, basilar artery deviation from the centerline was shown to be a risk factor for WMH laterality. We hypothesize that in these cases the basilar artery may supply the majority of blood for the two posterior cerebral arteries (22). Furthermore, basilar artery bending may create an asymmetrical perfusion in the two posterior cerebral arteries.

Our study is burdened by several limitations. Single-center design makes it possibly non-generalizable to the general population. TOF MRI images used in this study visualize the flow in the posterior intracranial circulation rather than assess the anatomical properties of the vessels. WMH burden was assessed based on only FLAIR MRI sequence, which may produce artifacts in the infratentorial regions. Finally, lack of automated quantitative measurements for WMH area limited the severity assessment.

In conclusion, we devised a repeatable and standardized technique to recreate and measure the morphology of the vertebrobasilar system. Our data suggests that the severity of WMH burden was mainly influenced by age and hypertension, while the localization of the WMH (or laterality) was mainly influenced by the vessel morphologies. Increased basilar artery curvature and increased infratentorial lateral WMH burden may signal inadequate blood flow and future cerebrovascular events. The effects of basilar artery morphology on WMH in the PCA region varied based on the circle of Willis variations. Further studies are needed to better understand these variations. Our findings might be used in the detection of intracranial hemodynamic disturbances and stroke prevention. However, further studies are needed to elucidate the associations between vertebrobasilar morphology and WMH severity.
